# Socio-economic determinants of malaria in tribal dominated Mandla district enrolled in Malaria Elimination Demonstration Project in Madhya Pradesh

**DOI:** 10.1186/s12936-020-03540-x

**Published:** 2021-01-05

**Authors:** Ravendra K. Sharma, Harsh Rajvanshi, Praveen K. Bharti, Sekh Nisar, Himanshu Jayswar, Ashok K. Mishra, Kalyan B. Saha, Man Mohan Shukla, Aparup Das, Harpreet Kaur, Suman L. Wattal, Altaf A. Lal

**Affiliations:** 1 Indian Council of Medical Research-National Institute of Research in Tribal Health, (ICMR-NIRTH), Jabalpur, Madhya Pradesh India; 2 Malaria Elimination Demonstration Project, Mandla, Madhya Pradesh India; 3 Directorate of Health Services, Government of Madhya Pradesh, Bhopal, India; 4 Indian Council of Medical Research, Department of Health Research, Ministry of Health and Family Welfare, New Delhi, India; 5 National Vector Borne Disease Control Program, Ministry of Health and Family Welfare, New Delhi, India; 6 Foundation for Disease Elimination and Control of India, Mumbai, Maharashtra India

**Keywords:** Socio-economic determinants, Malaria elimination, Tribal malaria, Rural households

## Abstract

**Background:**

Malaria is known as a disease of poverty because of its dominance in poverty-stricken areas. Madhya Pradesh state in central India is one of the most vulnerable states for malaria morbidity and mortality. Socio-economic, environmental and demographic factors present challenges in malaria control and elimination. As part of the Malaria Elimination Demonstration Project in the tribal district of Mandla in Madhya Pradesh, this study was undertaken to assess the role of different social-economic factors contributing to malaria incidence.

**Methods:**

The study was conducted in the 1233 villages of district Mandla, where 87% population resides in rural areas. The data was collected using the android based mobile application—SOCH for a period of 2 years (September 2017 to August 2019). A wealth index was computed along with analysis of the socio-economic characteristics of houses with malaria cases. Variables with significant variation in malaria cases were used in logistic regression.

**Results:**

More than 70% of houses in Mandla are *Kuccha* (made of thatched roof or mud), 20% do not have any toilet facilities, and only 11% had an annual income of more than 50,000 INR, which converts to about $700 per year. Households with younger heads, male heads, more number of family members were more likely to have malaria cases. *Kuccha* construction, improper water supply, low household income houses were also more likely to have a malaria case and the odds doubled in houses with no toilet facilities.

**Conclusion:**

Based on the results of the study, it has been found that there is an association between the odds of having malaria cases and different household variables such as age, gender, number of members, number of rooms, caste, type of house, toilet facilities, water supply, cattle sheds, agricultural land, income, and vector control interventions. Therefore, a better understanding of the association of various risk factors that influence the incidence of malaria is required to design and/or deploy effective policies and strategies for malaria elimination. The results of this study suggest that appropriate economic and environmental interventions even in low-income and poverty-stricken tribal areas could have huge impact on the success of the national malaria elimination goals.

## Background

Malaria is a global health problem and the World Health Organization (WHO) has estimated around 229 million cases of malaria and 409,000 deaths from malaria occurred worldwide in the year 2019 [1]. The incidence of malaria has declined globally from 80 cases per 1000 population at risk in year 2000 to 57 cases in the year 2019. Twenty nine countries contributed to 95% of the global malaria burden with 94% of the cases being contributed by the WHO African Region [1]. In India, malaria is a major public health concern. It contributed 86% of all malarial deaths in the WHO South East Asia region. India has the highest number of malaria cases (2% of global cases) and deaths (2% of malarial deaths) outside of the African sub-continent [1].

In India, malaria is reported from almost all states and union territories (UTs), but its transmission is not homogenous. The Indian states of Jharkhand, Chhattisgarh, Odisha, Uttar Pradesh, Gujarat, Madhya Pradesh and West Bengal together contribute more than 80% of the total malaria cases [2]. Although, about 89% of the country's population is at risk of malarial infection, but 80% of malaria cases are confined to areas consisting of 20% of the population residing in tribal, hilly, difficult and inaccessible area [3]. Approximately, 46% of total malaria cases, 70% of *Plasmodium falciparum* and 47% malaria deaths in India occur in tribal dominated areas [4].

Malaria epidemiology and its control are complicated by poverty as it is a dominant disease in poverty-stricken societies [5]. Madhya Pradesh (MP) is one of the vulnerable states in India and malaria control is complex because of its difficult geographical setup with the presence of many rivers and rivulets, deep valleys, hills and hillocks [6] with thick dense forest along with large tribal settlement (15% of India's tribal population) [7], poor socio-economic indicators [4] and inadequately understood socio-behavioural factors [8]. *Plasmodium vivax* and *P. falciparum* are the dominant species of malaria parasites in Madhya Pradesh. These parasites are highly seasonal in their distribution and it is mainly transmitted by *Anopheles culicifacies* and *Anopheles fluviatilis* [9, 10].

The Government of India has developed and launched a National Framework for Malaria Elimination (2016–2030) [11] and a National Strategic Plan (NSP, 2017–2022) [12], with a plan to eliminate malaria by 2027, three years ahead of global target [13]. Few studies have examined the association of socio-economic household factors affecting malaria incidence particularly in India [14–16]. The present study was undertaken in Mandla district, which is a tribal dominated district of MP to assess the role of different social, demographic, economic and household behavioural factors in malaria incidence.

## Methods

**Fig. 1:**
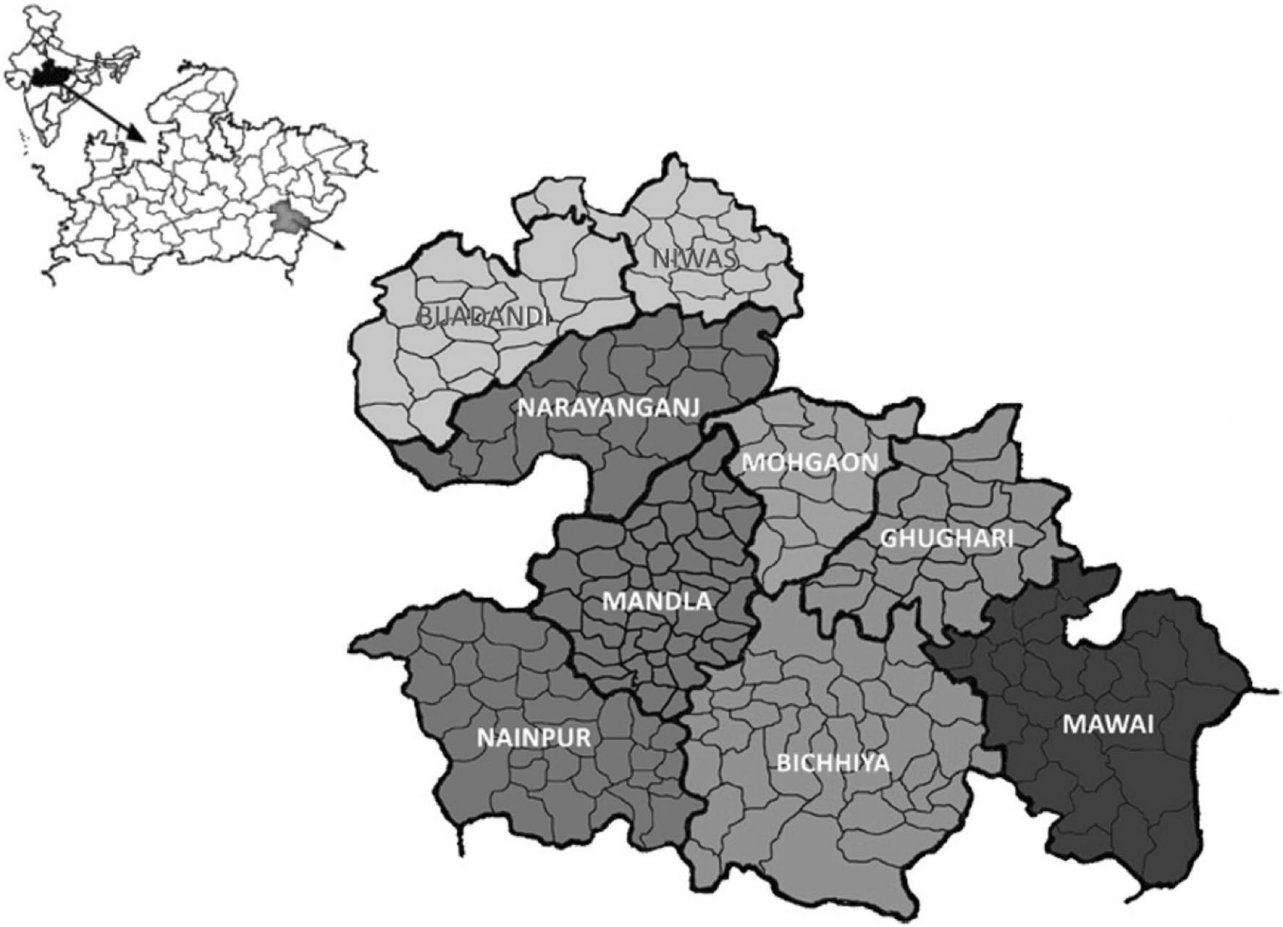
Geographical location of Mandla district

Study area and population: This study is a part of Mandla-Malaria Elimination Demonstration Project (MEDP), which is being carried out in the 1233 villages of Mandla district of Madhya Pradesh state in Central India. The district is located in the east—central region, an eastern district of Jabalpur division, which lies between the latitudes 22 ^°^02′ and 23 ^°^22′ North and longitudes 80 ^°^18′ and 81 ^°^50′ East, the district is at an altitude of 443 to 1100 m above the mean sea level (Fig. 1). The study district is a region of plains, hillocks and valleys with thick dense forest and Kanha National Tiger Reserve Park. Most of the villages are formed of many small hamlets and lies in undulating terrain with patches of forest. Many rivulets, perennial water streams pass throughout the district encircling many villages and creates numerous breeding sites for mosquitoes throughout the year. Agriculture along with forestry, animal husbandry and fisheries are the principal source of livelihood. The principal crops of the district are rice, wheat, *kodo* (*Paspalum scrobiculatum*), maize, gram, *tur* (pigeon pea), *masur* (lentil), *ramtil* (niger seed) and mustard [17–19].

The total area of Mandla district is 5,800 km [17], the population density of the district is 182 people per square kilometre. As per the Census 2011, the district had a total population of 1,054,905 residing in 250,146 households in 1233 villages and 9 development blocks About 87% of population was residing in the rural areas at the time of last 2011 census. And about 58% population was classified as scheduled tribes (ST) and another 4.6% as scheduled castes (SC) [17]. Mandla is one of the tribal dominated districts of the state and ethnic tribe ‘Gond’ and ‘Baiga’ live with other economically backward social groups in this area. These inhabitants are mostly poor, scantily clothed and spend most of their time outside the dwellings and sleep on the floor/cot in the *verandah* (porch) or out-of-doors. Domestic animals are often co-sheltered in the house [20]. Since 2017, the district’s malaria programme used alphacypermethrin 5% in IRS twice a year in areas with Annual Parasite Incidence (API) of 1 to 4.99. The LLINs were distributed in areas with API of 5 and above in 2017, and subsequently in areas with API of more than 2 in 2019. Neither IRS nor LLINs were provided in areas with less than 1 API.

### Data collection and analyses

The household data was collected through an android based mobile application named as SOCH—Solutions for Community Health workers [21]. The SOCH application is a good example of IT-based disease surveillance which allows surveillance, supply chain management, and workforce management tool. Some of the salient features of SOCH are—electronic surveillance and disease reporting systems; attendance management, intra-project communication and enablement of advance tour plans (ATPs) for the field staff; GPS tracing of field staff; built-in data validation protocols; online indents and requisitions and auto-deduction of stock; and a dashboard of key performance indicators. The application is being used in mobile surveillance and each household and study participants are assigned a unique ID. The baseline household information and surveillance data carried out during the last two years (Sept. 2017 to Aug. 2019) are used for this paper. The data is downloaded from the SOCH mobile apps server and transferred to IBM-SPSS-26 statistical software package (IBM Crop, Armonk, NY, USA).

A wealth index is computed adopting the commonly used methodology [22, 23] for demographic health surveys [24]. Households are given scores based on the number and kinds of consumer goods they own, housing characteristics such type of house, transport facility, source of drinking water, toilet facilities, number of rooms, agricultural land, cash crop, separate cattle shed, and annual income. All these variables are then dichotomized, and in total 27 dichotomous wealth proxy indicators used. The scores are derived using principal components analysis (PCA) to assign the indicator weights. Only the score of the first factors is used to represent the wealth index. The resulting sum is a standardized score with a mean of zero and a standard deviation of one. Household’s annual income is converted in to USD taking a conversion rate of one Indian rupee = 0.014 USD as on 27th February, 2020.

The socioeconomic characteristics of houses with at-least one malaria cases were compared with houses without any malaria cases. Chi-square test was used to study the association of variables with malaria case. The logistic regression technique was used and p < 0.05 was considered as significant. To study the association of household variables with malaria, logistic regression was used with binary outcome recoded (households with malaria case = 1, and households without malaria case = 0). Univariate and multivariate regression logistic regression models were used to compute unadjusted and adjusted odds ratios respectively. The odds ratios with their 95% confidence intervals (CI) were calculated to determine the relationship of socioeconomic household variables with the malaria.

The socio-economic household determinants of malaria were divided into three broad groups, viz. characteristics of the head of household (age, gender, and caste/social category of the head of households), housing characteristics (type of house construction, sources of drinking water and agricultural land and wealth index) and behavioural factors (mixed dwelling, i.e. co-residence with animals, IRS in houses and use of bed nets/LLINs). The household variables were categorized so that each category should have at least 30 households with malaria case.

All variables having significant variation in malaria cases across its categories (chi-square test, p < 0.05) were used in univariate logistic regression and unadjusted OR with 95% CI were computed. Multivariate analyses for variables significant in univariate analysis were performed by using logistic regression for all variables and a logistic regression with backward elimination (Wald) method was used to construct a model that includes all significant factors that remained significant in the presence of other significant variables. For all types of analyses, the unit of analysis was the household.

## Results

### The distribution of villages and population

Administratively district is divided into nine development blocks with 281 sub-centres and 1233 villages. The number of sub-centres varies from 23 sub-centres in Mohgaon block to 50 sub-centres in Mandla block. The villages per block also vary from less than 100 villages in Mohgaon and Ghughari blocks to 198 villages in Bicchiya block. Total 2,50,182 households were enlisted during baseline enrolment, and number of households varies from 16,642 households in Niwas to 52,674 households in Mandla block. On an average a village has 655 households in the district.

**Fig. 2:**
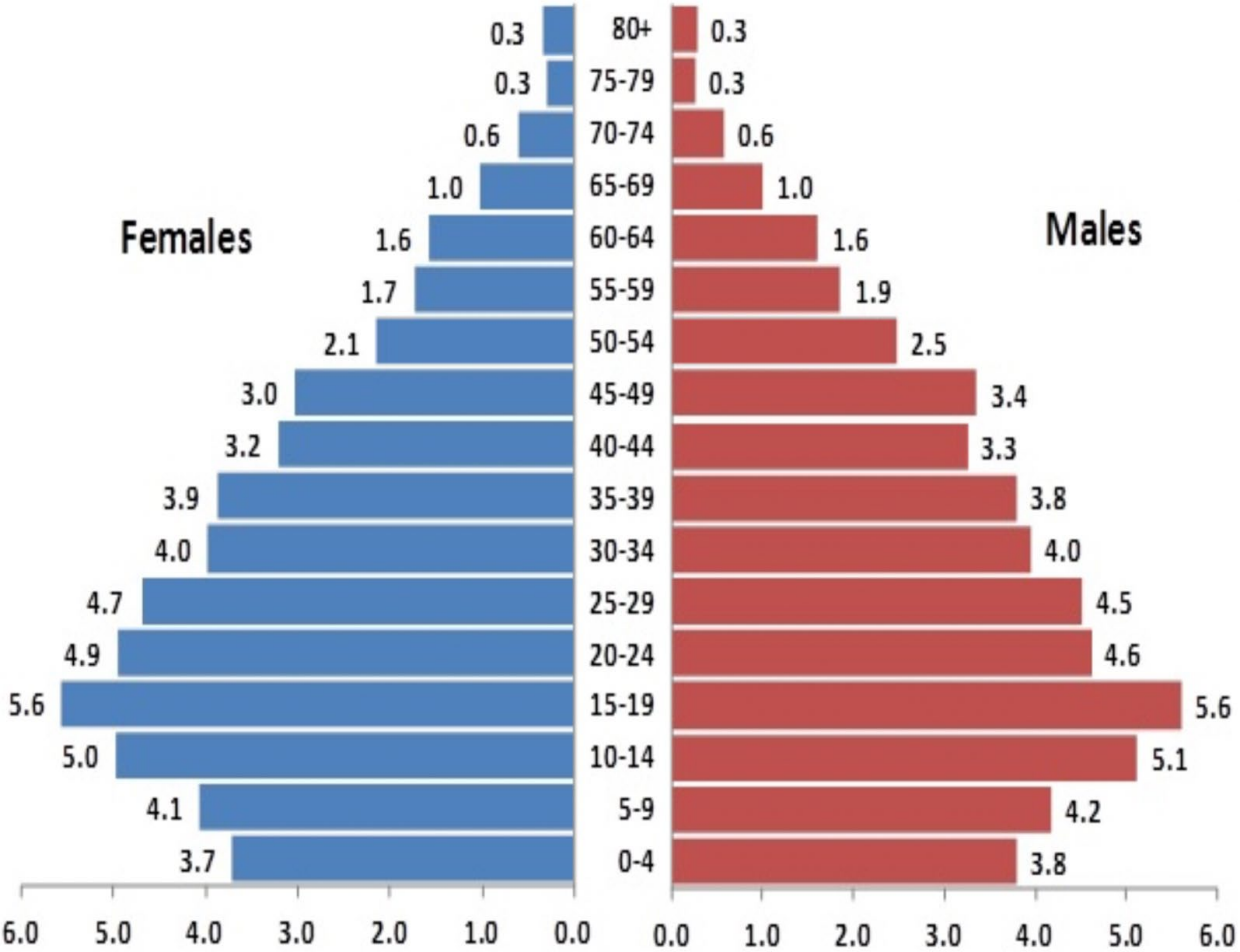
Age-sex pyramid of total population, Mandla

A total 11,43,126 people were enumerated in the baseline census conducted by MEDP project staff in the district. Four blocks, viz. Ghughari, Mawai, Bicchiya and Nainpur blocks were having more than 100,000 population, whereas all other blocks were having less than 100,000 population. The average household size was 4.6 persons per household in the district and it varies from 4.4 in Mandla block to 4.9 in Nainpur block (Table 1). Out of 11,43,126 enumerated persons, 49.8% are females and rest are enumerated as males. The total male female ratio is 992 females per 1000 males. About 8% of the total population is enumerated in both 0–4 years age group and 60 years or older age group (Fig. 2).

**Table 1:** Block-wise distribution of Sub-centres, villages, households and population in Mandla district

Blocks	No. of Sub centres	No. of villages	No. of HH	Average HH per village	Population	Average HH size	%ST Pop
MOHGAON	23	87	19559	804	89106	4.6	59.3
NARAYANGANJ	24	128	20183	1070	92782	4.6	72.4
NIWAS	24	100	16642	1188	77441	4.7	65.3
BIJADANDI	24	135	16796	267	79358	4.8	81.4
GHUGHRI	26	96	23868	381	108235	4.6	70.4
MAWAI	29	151	25338	688	113630	4.5	71.5
NAINPUR	35	159	36154	970	175876	4.9	51.4
BICHHIA	46	198	38968	140	175385	4.5	55.9
MANDLA	50	179	52674	668	231313	4.4	41.7
Total	281	1233	250182	655	1143126	4.6	59.3

Overall, about 59% of the total enumerated population is classified as ST in Mandla district and the proportion of ST population varies about 42% to 81% among the blocks of district.

Most of households have a male head (84.8%) and only 15.2% have female household heads. Further, 6.3% household heads are less than 30 years, whereas most of the household heads (72.8%) are between 30–59 years. The most of households (58.5%) belong to ST communities, 30.3% households belong to other backward castes (OBC) and another 8.5% belong to SC communities. About 35% households have 3–4 members in a family, and 49% households are having five or more members (Table 2).

**Table 2:** Characteristics of head of households

Characteristics	Numbers	Percent (%)
Age of head of household		
< 30 years	15696	6.3
30–44	88309	35.3
45–59	93763	37.5
60 +	52414	20.9
Gender of head of household		
Female	38038	15.2
Male	212144	84.8
Caste of head of household		
ST	146469	58.5
SC	21359	8.5
OBC	75911	30.3
Others	6443	2.7
Family size		
≤ 2	39712	15.9
3–4	88805	35.5
5 +	12665	48.6
Total	250182	100

In Mandla district, 97.5% households own their own house and more than 70% houses are *Kuccha* houses (made of thatched roof or mud). Only 21% houses are *pucca* houses (wall and roof made of bricks and cements) and 7% houses are semi-*pucca*, (wall or roof made of brick/cement). About 20% households do not have any toilet facilities, 61% and 17% households are having pit toilet flush toilet facility, respectively. Regarding availability of any means of transport at home, more than half households do not have any means of transport. About 30% households are using water from a well, another 34% and 29% households are using tube well and water taps for drinking water, while 6% households still fetch drinking water from stream/rivers.

About one third households reported annual income < 10,000 INR (~ $140) and 56% households have annual income between 10,000–50,000 INR (~ $140–700). Only 11% households reported annual income more than 50,000 INR (~ $700). About one-fifth houses are single room houses, half of the total houses have 2–3 rooms, and only 10% houses are having 5 or more rooms. About three-fourth houses possess some agriculture land and about 45% are involved in cash crop cultivation. About 64% households in the district have separate cattle shed, but only 7% households kept animals inside their houses. Every seventh household does not possess any bed net. Only 14.6% and 13.1% households owned one and two bed nets, respectively (Table 3).

**Table 3:** Characteristics of housing and availability of household amenities

Characteristics	N	%	Characteristics	N	%
Ownership of house			No. of rooms		
Rented	6229	2.5	1	55812	22.3
Own house	243953	97.5	2	53097	21.2
Type of house			3	72608	29.0
Kutcha	178598	71.4	4	43919	17.6
Semi-Pucca	17836	7.1	5+	24746	9.9
Pucca	53748	21.5	Agriculture land		
Type of toilet facility			No	59770	23.9
No Facility	49787	19.9	Yes	190412	76.1
Pit toilet	151780	60.7	Cash crops		
Flush toilet	42695	17.1	No	136341	54.5
Others	5920	2. 3	Yes	113841	45.5
Transport facility			Cattle shed		
No Facility	129136	51.6	No	88961	35.6
Bicycle	77793	31.1	Yes	161221	64.4
Motorcycle	41385	16.5	Cattle inside house		
Car	1868	0.8	No	231451	92.5
Source of drinking water			Yes	18731	7.5
Water stream /river	14837	5.9	Nos. of bed nets		
Well	76204	30.5	0	169651	67.8
Tube well	85661	34.2	1	36505	14.6
Tap water	73480	29.4	2	32863	13.1
Annual income			3+	11163	4.5
< 5 K	47800	19.1	Wealth index		
5–10 K	34701	13.9	Poorest	50309	20.1
10–25 K	71077	28.4	Second	49710	19.9
25–50 K	69505	27.8	Middle	50573	20.2
50 K +	27099	10.8	Fourth	49109	19.6
Total	250182	100	Least poor	50481	20.2

### Association of household variables with malaria

**Fig. 3:**
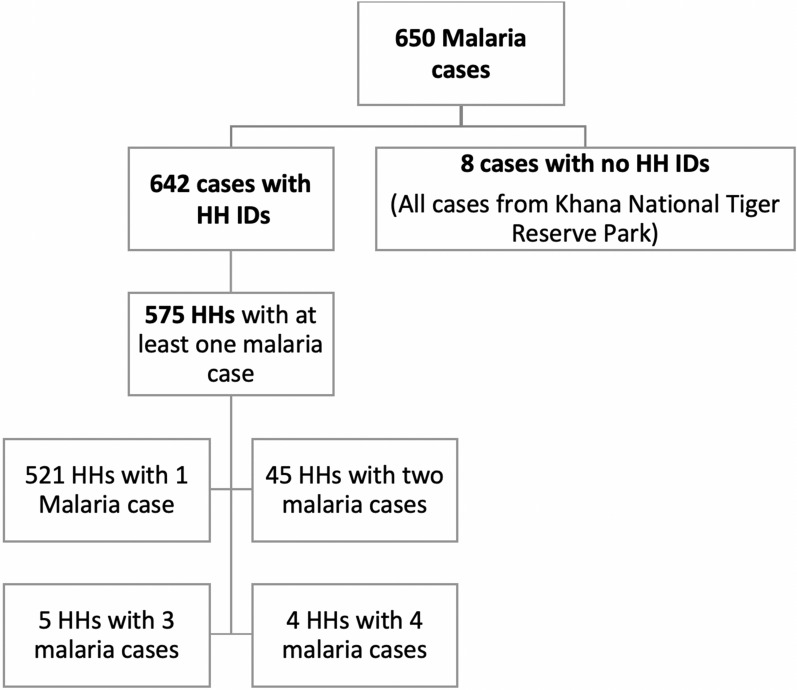
The distribution of malaria cases

During the last two years (Sept. 2017–Aug. 2019), 650 cases of malaria were identified and treated by MEDP project or Government programme staff. Out of these cases, 642 cases had unique household IDs, whereas, 8 cases were diagnosed from the Kanha National Tiger Reserve Park and do not have household information and unique IDs. All these 642 cases were from 575 households (Fig. 3). Thus, out of 2,50,182 households enrolled in MEDP project, only 575 households had a malaria case.

The univariate analysis shows that houses with younger (< 30 years) heads were significantly more likely to have a malaria case (OR = 1.76; 95% CI 1.21–2.55) compared to houses with older heads (60 + years). The relationship remains unchanged even after controlling for other household variables (AOR = 1.49; 95% CI 1.01–2.19). In the district, males are predominately reported as head (about 85% houses without, and 92% houses with malaria case) of households, and both univariate (OR = 2.22; 95% CI 1.63–3.03) and multivariate analyses (AOR = 1.76; 95% CI 1.28–2.41) shows that males headed houses are more likely to have a malaria case compared to female headed houses. Similarly, Scheduled tribe/caste houses are more likely to have malaria cases compared to other castes houses (AOR = 1.45; 95% CI 1.18–1.79). The analysis shows that household family size is significantly associated with malaria. The houses with 3–4 family members are more likely to have malaria case (AOR = 1.68; 95% CI 1.19–2.38) compared to houses with one or two members. Similarly, families having five or more members are also more likely have more malaria case (AOR = 2.27; 95% CI 1.63–3.17) compared to houses with fewer family members.

The house’s structure was also associated with malaria. The analysis shows that *Kuccha* houses are more likely to have a malaria case compared to semi-*pucca* or *pucca* houses (AOR = 1.49; 95% CI 1.181.88). Houses with no toilet facility are almost two times more likely to have a malaria case compared to houses with a flush toilet facility (AOR = 2.05; 95% CI 1.56–2.69). However, there is no significant difference between houses with flush toilet and a pit toilet facility. Similarly, more malaria cases were found in houses with no proper water supply (river/stream/pond/ well) (AOR = 1.22; 95% CI 0.98–1.51; p = 0.076) and houses with tube well water (AOR = 1.29; 95% CI 1.03–1.61) compared to houses with tap water. The univariate analysis also shows that households with less than 10,000 INR annual income are more likely (OR = 1.741; 95% CI 1.26–2.40) to have a malaria case compared to houses with 50,000 INR or more annual income. However, in multivariate analysis overall income shows a significant association with malaria, but individual categories loose its significance in the presence of other household variables.

Though malaria cases vary considerably by a number of rooms in the house (chi-square, p < 0.05), but univariate logistic regression analysis shows that chance of a malaria case does not vary significantly by number of rooms and thus variable is dropped in multivariate analysis, the households possessing agriculture land are significantly more prone to have a malaria case (AOR = 1.41; 95% CI 1.0–1.98) compared to houses without owning agriculture land. But houses engaging in the cultivation of cash crops have significant lower chances of having cases (AOR = 0.59; 95% CI 0.50–0.72) (Table 4). The wealth index shows that relatively better off houses are more likely to have malaria cases as compared to poor households. The households belonging to third, fourth and fifth quantile have significantly more chances to have a malaria case as compared to poorest houses. However, the wealth index lost its significance in the presence of other household variables.

**Table 4:** The association of household variables with malaria

Variables	% of HH with no malaria case	% of HH with malaria case	Unadjusted OR (95% CI)	Adjusted AOR (95% CI)
Age of head of HH				
< 30 years	6.3	7.3	1.76 (1.21–2.55)**	1.49 (1.01–2.19)*
30–44	35.3	37.6	1.60 (1.24–2.07)**	1.32 (1.01–1.72)*
45–60	37.5	41.2	1.66 (1.29–2.14)**	1.42 (1.20–1.84)**
60 +	20.9	13.9	–	–
Gender of head of HH				
Female	15.2	7.5	–	–
Male	84.8	92.5	2.22 (1.63–3.03)**	1.76 (1.28–2.41)**
Caste				
ST/SC	67.1	79.0	1.84 (1.51–2.25)**	1.45 (1.18–1.78)**
Others	32.9	21.0	–	–
Family size				
≤ 2	15.9	7.3	–	–
3–4	35.5	31.5	1.93 (1.38–2.69)**	1.68 (1.19–2.38)**
5 +	48.6	61.2	2.74 (1.99–3.78)**	2.27 (1.63–3.15)**
House type				
Kutcha	71.4	83.7	2.05 (1.65–2.56)**	1.49 (1.18–1.88)**
Semi Pucca/ Pucca	28.6	16.3	–	–
Toilet facility				
No facility	19.9	33.8	2.27 (1.74–2.97)**	2.05 (1.56–2.69)**
Pit toilet	60.7	50.8	1.13 (0.87–1.47)	1.17 (0.90–1.52)
Flush toilet	19.4	15.4	–	–
Source of drinking water				
Tap water	29.4	23.1	–	–
Tube well	34.2	33.6	1.24 (0.99–1.55)	1.29 (1.03–1.61)*
Others	36.4	43.3	1.51 (1.23–1.87)**	1.22 (0.98–1.51)
Annual income (Rs.)				
< 10 K	33.0	40.5	1.74 (1.26–2.40)**	1.31 (0.94–1.83)
10–50 K	56.2	51.8	1.31 (0.95–1.79)	1.00 (0.73–1.39)
50 + K	10.8	7.7	–	–
Rooms in household				
< 2	43.5	37.6	0.80 (0.61–1.07)	
3–4	46.6	51.8	1.04 (0.79–1.37)	
5+	9.9	10.6	–	
Ag. land				
No	23.9	16.2	–	–
Yes	76.1	83.8	1.63 (1.30–2.03)**	1.41 (1.10–1.81)**
Cash crop				
No	54.5	60.3	–	–
Yes	45.5	39.7	0.78 (0.67–0.93)**	0.59 (0.50–0.72)**
Cattle shed				
No	35.6	26.1	–	
Yes	64.4	73.9	1.56 (1.30–1.89)**	
Pet residing inside				
No	92.5	26.1	–	
Yes	7.5	73.9	0.63 (0.43–0.92)*	
HH covered in IRS				
No	90.6	83.5	–	–
Yes	9.4	16.5	1.91 (1.53–2.38)**	1.82 (1.45–2.28)**
Bednets				
No	67.9	48.2	–	–
≤ 2	27.7	40.5	2.06 (1.73–2.45)**	2.08 (1.74–2.48)**
3 +	4.4	11.3	3.58 (2.73–4.69)**	2.99 (2.26–3.96)**
Wealth index				
Poorest	20.0	12.0	–	
Second	20.0	14.8	1.39 (1.0–1.91)*	
Third	19.9	20.3	1.89 (1.40–2.54)**	
Fourth	20.1	27.3	2.48 (1.87–3.30)**	
Least poor	20.0	25.6	1.89 (1.41–2.54)*	
Total (N)	249607	575		

The analysis of behavioural and programme variable showed that house having separate cattle shed were more likely (OR = 1.56; 95% CI 1.30–1.89) to have a malaria case, whereas, houses with pet animals residing within the house were lesser to have a malaria case (OR = 0.63; 95% CI 0.43–0.92). But both of these variables lost significance in the presence of other household variables. The programmatic variables show that household covered in IRS reported more malaria cases. The houses covered in IRS were considerably more likely to have a malaria case (AOR = 1.82; 95% CI 1.45–2.28) against houses not covered in IRS. The houses with one or two bed nets (AOR = 2.08; 95% CI 1.74–2.48) and houses with three or more (AOR = 2.99; 95% CI 2.26–3.96) reported more malaria cases compared to households with no bed net (Table 4).

## Discussion

Malaria is a major public health problem in India despite being a both preventable and treatable disease. India recorded the highest decline (49%) in malaria cases in 2018 compared to 2017 [25] and from 2018 to 2019 was 17.6% [1]. The majority of malaria cases are reported from the eastern and central part of the country and from states which have forest, hilly and tribal areas. Madhya Pradesh state in the central India is one of the most vulnerable states to malaria because of the substantial population residing in the forest-fringe, foothills hard to-reach areas and having large populations of tribal ethnicity with poor awareness of disease prevention and access to treatment [23, 26].

Although malaria distribution is predominantly determined by the climatic and environmental factors affecting mosquito and malaria parasite reproduction and proliferation, however, malaria is also influenced by various socio-economic household factors [27–29]. In the present study, important associations between the occurrence of malaria cases and risk factors were observed. The study showed a strong association of malaria with age and gender of household head, social group, family size, type of housing, source of drinking water, availability of toilet facility, Agriculture land, cash crop production, and preventive measures.

The study shows that peoples from all age groups are affected by both the *Plasmodium falciparum* and *Plasmodium vivax* parasites, which are the two prevalent parasites in the study area [30]. This was different from observations in sub-Saharan Africa, where children under 5 years of age are most affected. The present study has revealed that even after controlling for other household levels socio-demographic, socio-economic and behaviour risk factors, the age of household head had a significant negative association with malaria. Several other studies have also reported the association of head’s age with the presence of malaria case in the household. These results were expected, as head’s age is a proxy of maturity and familiarity with symptoms, preventive methods and treatment of malaria [31]. The houses with younger heads are also more likely to have younger children in the household, having a higher risk of malaria patient at home [32]. The male head of households are more likely to engage in outdoor activities and females are relatively more engaged in indoor domestic activities. The division of labour as a result of gender roles may play a significant part in determining exposure to mosquitoes [33]. Many studies reported a similar risk for both genders [15, 16, 34]. However, some studies reported females to have a higher risk because they are primarily responsible for many household activities [35]and they start their day early and before dawn to perform household chores [36], but others reported males having greater occupation risk of contracting malaria [37, 38].

This study also demonstrated that considerably more scheduled tribe households had malaria cases compared others social group households. The reasons for it may be the lifestyle of tribal communities, compounded by mass poverty in these communities [26, 39]. Poor housing, engagement in outdoor activities, and outdoor sleeping habits are also common among rural and tribal communities and all these associated with malaria transmission in tribal areas [26, 40, 41]. Another highly significant socio-demographic variables observed in the study area was the family size. Families with 3–4 members and five or more members showed considerable higher chances of having a malaria case compared to a family with smaller families (≤ 2 members). Similar findings have also been reported by many other earlier studies, even though using different proxies, i.e. including the number of people in the house [15, 26, 31, 40] and the number of people per room [42]. This could be because large families are more likely to have younger children in the family which are a high risk group. The number of residents in a house also increase mosquito abundance as the olfactory cues for mosquitoes become stronger at crowding and attracts more mosquitoes [43].

The quality of house or material used for house construction is known to affect the entry of mosquitoes in dwelling places [16, 32, 42]. In the presence of other variables, *Kuccha* houses remained significant, and *Kuccha* houses have more malaria cases compared to semi-*pucca* or *pucca* houses. Many earlier studies have also drawn similar inference [16, 32, 40, 44, 45]. The mud housing is a threat to IRS done for vector control because of the practice of mud plastering soon after the spray [46].

The present study also demonstrated that houses with no toilet facility within the house are two-time more likely to have a malaria case compared to the house with having a flush toilet facility. The finding is in line of other studies which shows that poor sanitation facility as a significant risk factor [35, 47–49]. A recent study carried out in Pakistan shows that household with no toilet/non-hygienic toilet have lower risk of malaria, as hygienic toilets have greater chances of stagnant water, which may lead to mosquito growth [50]. The present study also revealed that dependence on outside water sources considerably increases the likelihood of having malaria infection. Similar findings were also documented by many other studies [35, 47, 49]. Households fetching water for domestic uses from tube-wells are higher risk of getting malaria infection. This could be as tube-wells are more likely to have stagnation water around it due to poorly maintained drainage channel [51], and may be surrounded by a large number of residents and usually congested with long queues, which increase mosquito breeding, biting and parasite transmission [48, 52] reported that piped water system significantly reduces the mosquito breeding sites.

The odds of *Plasmodium* infection also increased with a decrease in income [16, 53], the present study supported this finding; however, income lost its significance in the presence of other socio-economic variables. Occupation of household members has significant association with malaria. Cultivators and agricultural labourers are known to be at a higher risk through increased risk of contact with malaria vector at the field [40, 44]. People engaged in agriculture are at higher risk of malaria due to their outdoor sleeping, frequent movement in the forest seeking products, hunting, and protecting crops in field from animals [54] and inadequate treatment-seeking behaviour [55]. Another possibility is that, these people returned home after a tiresome day of work and may unknowingly take a deep sleep unaware of the vector bites and without taking protective measures [56]. In the present analysis, households having agriculture land showed significant association with malaria compared to houses without any agriculture land. However, study also demonstrated that household engaged in cultivation of cash crop are considerably having lower chances of having a malaria case. This may be because major cash crops of Mandla district are minor millets (Kodo-Kutki), Maize, Niger, Pigeon pea, Soyabean in Kharif crops season and Mustard, Lentil, and Chick pea in Rabi crops season are less water intensive compared to major foodgrain crops, Paddy in Kharif and Wheat in Rabi crops [18, 19]. The earlier study also showed that households having irrigated land or involved in rice cultivation have higher chances of malaria [57].

As household income is difficult to measure in low-income settings because of multiple sources of income, and seasonal or annual variation in income [58]. So, many researchers have used composite wealth index as proxy of household income or socio-economic status (SES). The relationship between malaria disease and poverty often described as a vicious cycle, whether malaria infection is a consequence of or a cause for low household socioeconomic status has been debated for decades [59]. Many studies showed a significant negative relationship of wealth index with malaria, i.e. the poorest households have significant more malaria cases compared to relatively better-off households [45, 50, 60]. However, the present study showed a contradictory finding, i.e. better off households have more malaria cases compared to poorest houses. The wealth index lost its significance in the presence of other variables, and could not be included in final model. This is similar to findings to studies carried out in Tanzania [60] Ethiopia [32] and Kenya [61], which shows that SES had no association with malaria infection. Worrall et al*.* in a systematic review of nine studies, revealed that two studies found a significant positive relationship between poverty and malaria, four studies found no significant relationship and three studies demonstrated mixed results [55]. The association observed between wealth index and malaria in the present study is very similar to the finding of a study carried out in southern Nigeria, which reported more malaria among better off SES compared to poor [62]. The positive association of SES index with malaria may be because the variables included in the wealth index, such as house type, source of water, toilet facility are also independently malaria risk factors.

Several studies have documented the effect of cattle near to or in the house in relation to malaria is inconsistent [47, 63]. Some studies showed that keeping cattle in the house was a risk factor for occurrence of malaria [47, 64, 65], while other study does not find any such relationship [48]. The present study shows that likelihood of having malaria in households having separate cattle shed and pets residing outside of houses are higher. This shows that cattle rearing close to human habitations act as a Zoo-prophylaxis. However, in the presence of other socio-economic variables, both variables lost their significance and could not be included in the final model. Thus, study does not show any conclusive relationship with pets’ co-residence or keeping animals outside in separate cattle shed with malaria.

The uses of malaria control measures, such as insecticide residual spray (IRS) and use of insecticide-treated nets (ITN)/long-lasting insecticide-treated nets (LLIN) or other preventive measures significantly reduced the chances of getting malaria infection. An earlier study carried has documented the role of these measures in bringing down malaria cases [16]. However, the findings of the present study are in contrast to the previous study. This observation may be due to the fact that IRS was implemented in high-prevalence areas of the district (API 1–4.99). Similarly, possession of bed nets significantly increased the chances of having malaria, which could also be because LLINs were distributed initially in areas with > 5 API in year 2017, and subsequently in areas with > 2API in year 2019. Similar, findings were reported by some other studies in India [16] and Africa [66, 67]. Malaria occurrence was found to be higher among those using LLINs in Assam and torn and improperly used LLINs allow mosquitoes to enter and bite the user [16].

The National Vector Borne Disease Control Programme (NVBDCP) of India carries out vector measures throughout the country, and vector control strategy in India is primarily based on the two rounds of IRS and free distribution of LLIN bed nets based on the area’s API. Houses covered in IRS and possessing LLIN bed nets are from highly malaria endemic areas. Further, this study has documented that at the start of this study (2017) about 68% households do not have bed-nets. Therefore, poor distribution/availability of bed-nets in this high-prevalence district may have been a major factor in continued transmission of malaria in this district. Furthermore, utilization of available bed nets remains an issue, misuses of bed nets is well-documented in tribal dominated areas of Madhya Pradesh [26, 68]. But finding suggests that even with these vector control measures, households from high endemic areas have higher odds of having malaria infection compared to households from lower endemic areas in the district.

In conclusion, this study has revealed that there is an association between the odds of having malaria cases and different household variables such as age, sex, number of members, number of rooms, caste, type of house, toilet facilities, water supply, cattle sheds, agricultural land, income, and vector control interventions. Complementary vector control and case management interventions are needed to further reduce malaria transmission. This study reveals that in tribal areas where poverty is rampant, the use of preventive means is not universal, which maybe the reason for sustained transmission of malaria. Finally, the results of this study suggest that appropriate economic and environmental interventions even in low-income and poverty-stricken tribal areas could have huge impact on the success of the national malaria elimination goals.

## Data Availability

We have reported all the findings in this manuscript. The hardcopy data is stored at MEDP Office in Mandla, Madhya Pradesh and Indian Council of Medical Research -National Institute of Research in Tribal Health (ICMR-NIRTH), Jabalpur, Madhya Pradesh. Softcopy data is available on the project server of MEDP hosted by Microsoft Azure. If anyone wants to review or use the data, they should contact. Dr. Altaf A. Lal. Project Director – Malaria Elimination Demonstration Project, Mandla. Foundation for Disease Elimination and Control of India, Mumbai, India 482,003. E mail: altaf.lal@sunpharma.com.

## Abbreviations

CI: Confidence Interval
IRS: Indoor Residual Spray
ITN: Insecticide-Treated Nets
LLIN: Long-Lasting Insecticide-treated Nets
MEDP: Malaria Elimination Demonstration Project
MP: Madhya Pradesh
OBC: Other Backward Castes
PCA: Principal Components Analysis
SC: Scheduled Castes
ST: Scheduled Tribes
UT: Union Territory
